# ECMO to CPB: A single circuit approach

**DOI:** 10.1051/ject/2025035

**Published:** 2025-12-17

**Authors:** Jeffrey Zalfa, Daniel Duncan, Paul Kerins

**Affiliations:** 1 Nemours Children’s Hospital 1600 Rockland Road Wilmington DE 19803 USA

**Keywords:** ECMO, CPB, Perfusion, Pediatric heart surgery, Congenital heart disease

## Abstract

*Background*: Veno-arterial extracorporeal membrane oxygenation (VA-ECMO) and cardiopulmonary bypass (CPB) are used during pediatric heart surgery to provide cardiopulmonary support to patients as they undergo and recover from surgical procedures. On occasion, CPB and extracorporeal membrane oxygenation (ECMO) circuits are used within the same surgical period. In this technique article, we report on our experience using an ECMO circuit with the addition of a cardiotomy reservoir to convert to CPB. *Methods*: Patients on VA-ECMO were converted to CPB by the splicing of a cardiotomy reservoir and ECMO circuit. *Results*: Seven patients underwent conversion from VA-ECMO to CPB with a total of eight procedures. Mean nadir hematocrit on CPB was 31.1% ± 6.06. Mean blood product usage on CPB was 238 ± 155 mL. All the patients were decannulated from CPB or ECMO. Conclusion: Conversion from VA-ECMO to CPB with the use of the same circuit is an effective technique for congenital heart patients on VA-ECMO who require surgical intervention with CPB.

## Introduction

The use of extracorporeal membrane oxygenation (ECMO) has been reported since the 1970s and is used today for the rescue of acute cardiac or respiratory failure [1–3]. Veno-arterial extracorporeal membrane oxygenation (VA-ECMO) is used in congenital heart disease patients as a bridge-to-recovery, bridge-to-transplant, or preoperative support in the intensive care unit (ICU) [1, 3–7]. Surgical intervention occasionally necessitates the need for transition from VA-ECMO to cardiopulmonary bypass (CPB) [6–8]. In this technique article, we report on our recent experience of converting patients from VA-ECMO to CPB by adding a cardiotomy reservoir with an ECMO circuit.

There are important differences between VA-ECMO and CPB that preclude a successful operation on VA-ECMO alone. ECMO circuits consist of a membrane oxygenator, cannulas, and circuit tubing, whereas CPB has the addition of a cardiotomy reservoir and cardiotomy suction [6–8]. The ability for the surgeon to aspirate shed surgical blood for rapid reinfusion back into the circuit is crucial to preserving circulating volume, especially for the neonatal or infant patient who has a total blood volume of a few hundred milliliters [9, 10]. In addition, a cardiotomy reservoir allows the ability to decompress the heart, apply ventricular venting, and decrease blood volume during periods of deep hypothermic circulatory arrest (DHCA) [6–8].

There are considerable drawbacks to the use of multiple extracorporeal circuits on the pediatric patient. The priming volume of a pediatric ECMO circuit is approximately 250 mL, which has an outsized dilutional effect on the diminutive blood volume of the neonate or infant [9–11]. Repeated blood donor exposure used in the priming of the extracorporeal circuits is also linked with nosocomial infection, increased ventilator time, and mortality [12, 13]. Moreover, the inflammatory response caused by repeated exposure to foreign surfaces further contributes to physiologic derangements, coagulopathy, and morbidity [14, 15]. Using this technique, the authors intended to lessen the use of allogenic blood products, preserve the blood in the ECMO circuit, and reduce foreign surface exposure.

## Materials and methods

The Institutional Review Board of Nemours/Alfred I. duPont Hospital for Children determined this study to be exempt from further review on October 30, 2024 (IRB#2256017).

The ECMO system consisted of a ¼″ Maquet BioLine coated circuit (Getinge, Rastatt, Germany) and an AMG PMP Infant Oxygenator (Abbott Laboratories, Chicago, Illinois, USA) mounted on a Sorin S5 heart-lung machine (LivaNova, London, UK). The circuit was primed with Plasma-Lyte crystalloid solution (Baxter, Deerfield, Illinois, USA) and available on standby for emergent initiations. Prior to initiation, the circuit was circulated at 37 °C while blood products and cannula were obtained. The circuit was primed with a portion of one unit of packed red blood cells (PRBCs) or reconstituted whole blood (RWB), depending on urgency and availability. Following blood prime, 100 IU/kg of heparin, 10 mEq of sodium bicarbonate, and 300 mg of calcium chloride were administered into the circuit and allowed to recirculate. Flow rates were determined by the surgeon within the range of 50–100 cc/kg/min. Continuous heparin infusions were started after activated clotting times were measured at 180–220 s. Following ECMO initiation and patient stabilization, the patient was brought to the operating room (OR) when surgery was approved. The patient was transported from the ICU to the OR with a team of anesthesiologists, perfusionists, ECMO specialists, and OR nurses.

### Cardiotomy reservoir to the ECMO circuit

The cardiotomy reservoir was assembled with a Terumo Capiox FX05 Oxygenator (Terumo Cardiovascular Systems, Elkton, MD, USA), ¼″ connectors, ¼″ wye connectors and ¼″ tubing (LivaNova, London, UK). First, the oxygenator was removed and discarded, leaving a stand-alone reservoir. Then, the ¼″ tubing was connected to the inlet and outlet of the reservoir to create a continuous loop. Two ¼″ wye connectors were cut into the loop, approximately 4″ from the inlet and outlet of the reservoir. ¼″ tubing was connected to create a recirculation line. Approximately 6″ of ¼″ tubing was connected to the other side of each ¼″ wye. A ¼″ connector was inserted at the end of this tubing, which would be used later to connect to the ECMO circuit.

The reservoir and tubing were primed with fresh whole blood (FWB) or RWB using a blood transfusion filter (Haemonetics, Boston, MA, USA), and the level sensor was engaged. The recirculation line was primed and clamped for the duration of the procedure. 200 IU/kg of heparin and 10 mEq of sodium bicarbonate were added to the prime. The cardiotomy manifold inlet was connected to a three-way stopcock on the post-oxygenator pigtail of the ECMO oxygenator and kept closed. A CardioQuip MCH-1000(i) heater-cooler (College Station, TX, USA) was connected to the ECMO oxygenator and set to the current patient temperature. Sterile towels were placed underneath the cardiotomy lines. The ECMO lines and cardiotomy lines were prepped with betadine solution in the areas that were determined to be cut and connected. Two perfusionists obtained sterile clamps and scissors and donned sterile gloves. A time-out was called in the OR, stating the steps of the procedure and the role of the personnel involved. It was communicated that the patient would be off extracorporeal support for approximately 10–20 s.

**Figure 1:**
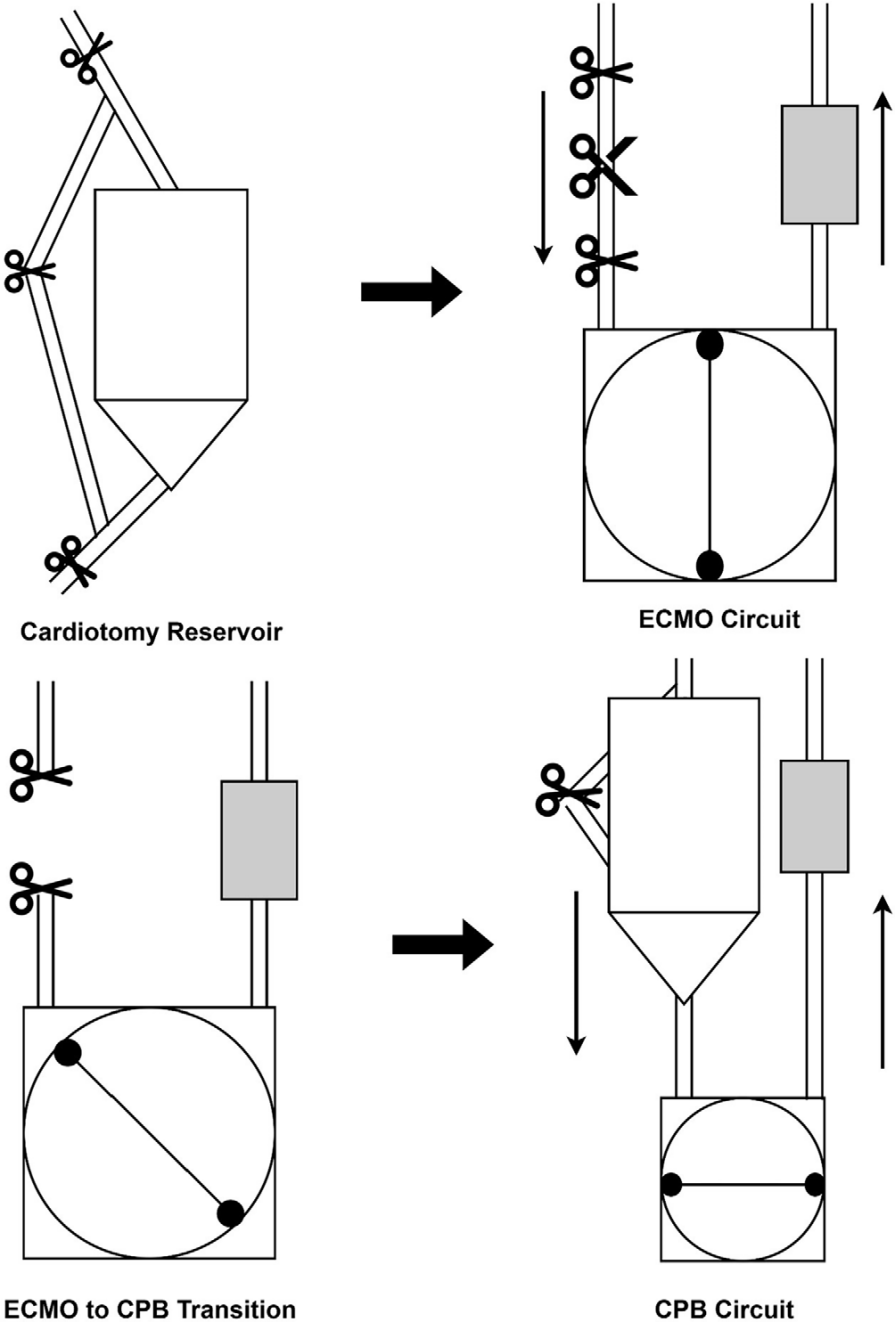
ECMO to CPB transition.

Following the time-out, the ECMO circuit was stopped. The venous limb of the ECMO circuit was clamped twice and cut in between the clamps using sterile technique. The patient limb of tubing was connected to the ¼″ connector that drains to the inlet of the cardiotomy reservoir. The circuit limb, which flows into the roller-pump, was wet-to-wet connected to the ¼″ connector of the outlet of the reservoir. Once the connections were deemed secure, all clamps were released, forward flow was re-established, and CPB was initiated (Fig. 1). The three-way stopcock on the post-oxygenator pigtail was opened and was the source of manifold shunt flow and arterial pressure monitoring. The heater-cooler was set to the desired temperature as directed by the surgeon, and the remainder of CPB was conducted normally.

## Results

Seven patients underwent transition from VA-ECMO to CPB, with one patient undergoing twice, for a total of eight procedures. Mean age was 88 ± 92 days. Five of the patients were male, and two of the patients were female. Mean height was 53.2 ± 6.16 cm, and median weight was 3.4 kg (IQR = 2.89). Mean body surface area (BSA) was 0.23 ± 0.06 m^2^. Three out of the seven patients had a pre-operative diagnosis of Tetralogy of Fallot, one had Truncus Arteriosus, two had Total Anomalous Pulmonary Venous Connection, and one had Aortic Arch Hypoplasia and Atrioventricular Canal. All the patients were decannulated from ECMO or CPB. Demographic data is listed in Table 1.

**Table 1:** Demographics.

Demographics	Age (days)	Sex (M/F)	Height (cm)	Weight (kg)	BSA (m^2^)	Diagnosis	Decannulation (Y/N)
Patient 1 (a)	126	Male	58	6.4	0.29	Tetralogy of Fallot	Y
Patient 1 (b)	127	Male	58	6	0.29	Tetralogy of Fallot	Y
Patient 2	179	Male	50	3.4	0.21	Truncus Arteriosus	Y
Patient 3	2	Female	53	3.3	0.21	TAPVC	Y
Patient 4	3	Male	52	3.4	0.21	TAPVC	Y
Patient 5	240	Female	62.5	6	0.31	Aortic Arch Hypoplasia, AV Canal	Y
Patient 6	10	Male	43	2.54	0.16	Tetralogy of Fallot	Y
Patient 7	22	Male	49	2.44	0.18	Tetralogy of Fallot	Y
Mean ± SD	88 ± 92		53.2 ± 6.16	4.19 ± 1.66	0.23 ± 0.06		
Median (IQR)	74 (132)		52.5 (8.25)	3.4 (2.89)	0.21 (0.09)		

a: 1st conversion, b: 2nd conversion, BSA: body surface area, TAPVC: total anomalous pulmonary venous connection, AV: atrioventricular, SD: standard deviation, IQR: interquartile range.

Mean pre-CPB hematocrit (HCT) was 37.9% ± 6.01, mean CPB nadir HCT was 31.1% ± 6.06, and mean post-CPB HCT was 40.1% ± 4.12. Mean blood product usage was 238 ± 155 mL. Mean time from initial ECMO cannulation to CPB was 7,630 ± 6,529 min. Median CPB time was 97 min (IQR = 62.8). Three out of the eight procedures required an aortic cross-clamp and DHCA. For these procedures, the mean aortic cross-clamp time was 71.7 ± 59.4 min and the mean DHCA time was 70 ± 56.6 min. Operative data is listed in Table 2.

**Table 2:** Operative data.

Operative data	Pre HCT (%)	Nadir HCT (%)	Post HCT (%)	Blood products (mL)	ECMO to CPB (min)	CPB (min)	XC (min	DHCA (min)
Patient 1 (a)	28	28	38	150	12,175	41	n/a	n/a
Patient 1 (b)	43	39	47	150	960	91	n/a	n/a
Patient 2	37	33	40	90	9,255	77	n/a	n/a
Patient 3	45	20	39	550	2,964	336	140	135
Patient 4	45	38	45	292	940	126	43	43
Patient 5	36	31	38	195	19,448	145	n/a	n/a
Patient 6	35	28	40	357	4,156	103	32	32
Patient 7	34	32	34	120	11,141	23	n/a	n/a
Mean ± SD	37.9 ± 6.01	31.1 ± 6.06	40.1 ± 4.12	238 ± 155	7,630 ± 6529	118 ± 97	71.7 ± 59.4	70 ± 56.6
Median (IQR)	36.5 (8.75)	31.5 (6.25)	39.5 (3.25)	173 (166)	6,706 (8937)	97 (62.8)	43 (54)	43 (51.5)
Min	28	20	34	90	940	20	32	32
Max	45	39	47	550	19,448	336	140	135

a: 1^st^ conversion, b: 2^nd^ conversion, HCT: hematocrit, ECMO: extracorporeal membrane oxygenation, CPB: cardiopulmonary bypass, XC: aortic cross-clamp, DHCA: deep hypothermic circulatory arrest, SD: standard deviation, IQR: interquartile range.

## Discussion

The pediatric heart patient’s size makes them increasingly susceptible to dilutional anemia and coagulopathy. The prime volume of an ECMO or CPB circuit is nearly equal to the circulating volume of the patient, which potentially requires copious use of blood products and hemofiltration. While there is still the need to prime the cardiotomy reservoir with blood, we attempted to lessen the total amount of blood products administered, and importantly, save the blood that is in circulation. In addition, we also save any FWB or RWB that was spiked but not used in the prime and offer it to anesthesia for post-operative reinfusion.

A considerable contributing factor to our success with this technique is our institutions’ use of FWB and RWB. FWB is a single donor blood product that is not separated into components and contains red blood cells, plasma, and platelets. RWB is a mixture of PRBCs and fresh frozen plasma from multiple donors that is designed to mimic FWB, albeit with no platelets. If FWB is unavailable, our blood bank will provide RWB as a replacement. The use of these balanced blood products allows us to prime the cardiotomy with one constituent and initiate bypass without the need for hemofiltration beforehand. We simply prime the cardiotomy with a portion of blood product, add our prime medications, and begin the procedure. This alleviates the need for additional equipment and simplifies the workflow. Since we use FWB or RWB as our routine priming blood product, we can complete this technique without forgoing our normal protocol.

Our ECMO pumps are built with the ability to institute this process rapidly. Each of our ECMO roller pumps has two additional pumps mounted that can be used for cardiotomy suction and cardioplegia delivery. We routinely use a crystalloid-based cardioplegia, so there is no requirement to connect our cardioplegia circuit to the oxygenator. When we are made aware that we will be transitioning from VA-ECMO to CPB, we need only transfer ancillary equipment such as manifold holders, venous assisted drainage suction, and cardioplegia disposables. This setup is beneficial to be able to implement this procedure in a timely, efficient, and safe manner.

Of note, if we need to return to VA-ECMO from CPB, the recirculation line provides us with the ability to transfer seamlessly back to VA-ECMO. We simply unclamp the recirculation line and clamp the venous reservoir inlet and outlet lines. We can then cut out the venous reservoir and re-attach those lines to create a loop in the venous inflow line. If we had to re-institute CPB again, we could insert another reservoir into this loop without discontinuing extracorporeal support.

If there is any indication of circuit thrombosis or oxygenator failure, our team will forgo using this technique. We run serial blood gases and hematologic assays on our ECMO patients as well as visual inspection of circuit components. If there is any indication that the circuit is compromised, we would elect to use either another ECMO or CPB circuit.

## Limitations

This study is limited by the fact that it does not compare a group of patients who were transitioned from VA-ECMO to our normal CPB circuit. Furthermore, it does not test whether the amount of blood product we used was different from what it would have been if we converted to our normal CPB circuit. The acuity and infrequency of this patient population make it a difficult population to study, and this article is not definitive on the best approach to handle these clinical situations. Further study would be necessary to demonstrate the overall effectiveness of this approach compared to complete circuit exchange.

## Conclusion

In this report, we describe our experience with this technique in a variety of surgical situations, from brief bypass runs to several hours with multiple periods of aortic cross-clamp and DHCA. In our experience, we were able to convert to CPB without losing any of the additional functions that are normally provided. We found this to be an effective way to provide two different forms of cardiopulmonary support using the same circuit components. Further research is required to ascertain whether this technique is advantageous as compared to complete circuit exchange or other novel techniques.

## Data Availability

All data is presented in this manuscript.
